# Identification of hepatitis B virus genotype I in Thailand

**DOI:** 10.1002/jmv.25346

**Published:** 2018-11-08

**Authors:** Vera Holzmayer, Robert Hance, Patricia Defechereux, Robert Grant, Mary C. Kuhns, Gavin Cloherty, Mary A. Rodgers

**Affiliations:** ^1^ Abbott Laboratories, Infectious Disease Research, Abbott Park Illinois; ^2^ University of California San Francisco San Francisco California

**Keywords:** hepatitis B virus, recombinant virus, recombination, virus classification

## Abstract

The rare hepatitis B virus genotype I (HBV‐I) classification includes complex A/G/C/U recombinants identified amongst the individuals from China, India, Laos, and Vietnam. Herein we report the first HBV‐I specimen from Thailand, with detectable HBsAg despite a 10–amino‐acid truncation. This HBV‐I genome has a similar recombinant pattern to reference strains, including a C region that branches basal to references, suggesting a premodern era recombination event gave rise to HBV‐I. With an average sequence divergence from other genotypes ranging from 7.66% (standard deviation [SD], 0.42%; C) to 14.27% (SD, 0.31%; H), this new genome supports the HBV‐I classification as a unique genotype.

## 1 INTRODUCTION

Despite introduction of a vaccine for hepatitis B virus (HBV) in 1982, the global burden of HBV remains high, with an estimated 257 million chronic infections globally, of which more than 90% remained undiagnosed in 2015.^1^ Accurate diagnosis of HBV is a critical initial step in caring for patients with HBV and preventing new transmissions; however, the diversity of HBV sequences presents a challenge for diagnostic tests.^2^ It has been estimated that HBV was first introduced into the human population approximately 33 600 years ago,^3^ and since then it has evolved to nine unique genotypes (A‐I) and a growing list of more than 30 subgenotypes.^4^, ^5^, ^6^ This diversity is largely driven by the error‐prone viral reverse transcription polymerase, which lacks proofreading abilities and intergenotypic recombination.^6^, ^7^ HBV genotypes and subgenotypes are unevenly distributed, with each HBV genotype predominating in unique regions of the world.^4^ In particular, HBV genotype I (HBV‐I) has been found in China,^8^, ^9^, ^10^, ^11^ India,^12^, ^13^ Laos,^14^ and Vietnam,^15^, ^16^, ^17^ with 39 unique sequences confirmed cases in Asia to date.^5^ In addition to these cases, HBV‐I strains have also been sequenced from Vietnamese immigrants living in Canada^18^ and France,^19^ indicating that a more widespread distribution may exist for HBV‐I outside of the Asia. Although HBV‐I sequences branch together in a phylogenetic tree, recombination analysis has determined that HBV‐I sequences are complex recombinants of genotypes A, C, G, and unclassifiable sequences with exact breakpoints varying between individual strains.^7^, ^8^, ^12^, ^14^, ^15^, ^16^, ^18^, ^20^ Classification of these strains as HBV‐I or A/C/G/U recombinants is a topic of debate^7^, ^8^, ^21^, ^22^ since the classical definition of a unique HBV genotype is at least 8% intergenotypic divergence, yet HBV‐I strains are differ from the closest relative, genotype C, by only 7 to 7.9%.^8^, ^12^, ^14^, ^15^, ^18^, ^22^ Thus, in additional, putative HBV‐I genome sequences will be required to determine the appropriate classification for these unique sequences.

HBV coinfection with human immunodeficiency virus (HIV) puts patients at higher risk for severe liver disease, making prevention of such infections a priority.^1^ The recent evaluation of HIV pre‐exposure prophylaxis (PrEP) regimens by several groups has led to the successful implementation of PrEP programs amongst high‐risk populations.^23^, ^24^ Emerging evidence supports the safe use of PrEP amongst HBV carriers.^25^ In a recent assessment of the safety of PrEP for patients with active HBV infection, serial blood samples were collected from 12 participants who had chronic HBV infections at the time of enrollment to monitor their infections with and without PrEP treatment during the iPrEx study.^25^ HBV sequences obtained from these individuals indicated that resistance mutations did not develop amongst the participants on PrEP, supporting the safe use of PrEP for chronic HBV patients.^25^ The samples were collected from diverse geographical regions (United States, Thailand, Peru, Ecuador, and South Africa), although the HBV genotypes for these infections were unknown. To evaluate the HBV diversity encompassed within the iPrEx study, we characterized the sequences generated from serial blood draws of the chronic HBV participants and identified an HBV‐I infection among them, which we characterized in depth to expand the known diversity of the genotype I classification.

## 2 MATERIALS AND METHODS

### 2.1 Samples

The sample cohort has been described previously,^25^ and a complete diagnostic profile of the HBV‐I infection time course can be found in Figure 1C of Solomon et al.^25^ Sequences were generated from leftover cryopreserved plasma collected at the screening visit (day 12), 0, 79, 112, 251, and 336 days timepoints. All specimens were collected with informed consent from donors as approved by the University of California San Francisco IRB.

**Figure 1:**
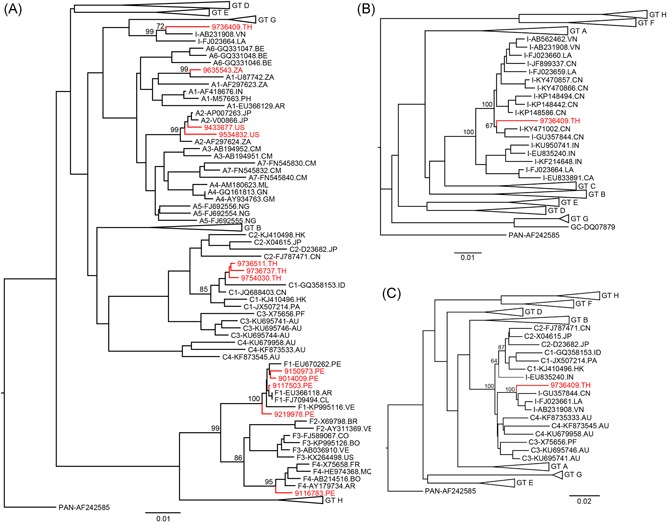
HBV phylogenetic trees. Neighbor‐joining phylogenetic trees are shown for the 798 nucleotide surface antigen/reverse transcriptase (SRT) fragment (genome coordinates 204‐1001 nucleotides) (A), the 3215 nucleotide complete genomes (B), and the nucleotide position 1500 to 2900 region of the HBV genome (C). Branches without any sample sequence are depicted as triangles. Sample sequences are indicated in red and references are in black. Individual strains are labeled with their classification, GenBank accession number, and the two‐letter country code for their source. Bootstrap values are provided for nodes relevant for sample classification. The tree in (B), is representative of a larger tree that includes a comprehensive set of 37 HBV‐I reference sequences from Canada, Vietnam, China, Laos, and India. Two‐letter codes at the end of each sequence label indicate the source country. GT, genotype; HBV, hepatitis B virus; HBV‐I, hepatitis B virus genotype I

### 2.2 Sequencing

Subgenomic sequences were generated for the surface antigen/reverse transcriptase (SRT) region as previously described.^26^ The HBV‐I genome was amplified in two overlapping fragments using Adventage‐2 polymerase reagents (Clontech, Palo Alto, CA). Primer pairs for the S‐core fragment were HBV‐238F (5′‐ATACCACAGAGTCTAGACTCGTGGTGGACT‐3′, nucleotides (nt) 236‐265)/ HBc‐2382R (5′‐CGTCKGCGAGGCGAGGGAGTTC‐3′, nt 2382‐2403) for the first‐round PCR and HBrt‐295F (5′‐GTGTCYTGGCCWAAATTCGCAGTCCC‐3′, nt 295‐320)/HBc‐2298R (5′‐TGTKGATARGATAGGGGCATTTGGTGGTC‐3′, nt 2298‐2336) for the nested PCR. Primers for the core‐pol fragment were HBc‐1776F (5′‐ GGAGGCTGTAGGCATAAATTGGTCTG‐3′, nt 1776‐1801)/ HBrt‐1253R (5′‐ GCAGTATGGATCGGCAGAGGAG‐3′, nt 1253‐1274) for the first‐round PCR and HBc‐1882F (5′‐ CCTTGGGTGGCTTTRGGRCA‐3′, nt 1882‐1901)/ HBrt‐1186R (5′‐ CCAGTGGGGGTTGCRTCAGC‐3′, nt 1186‐1205) for nested PCR. First‐round and nested second‐round amplifications consisted of preincubation at 95°C for 1 minute, 40 cycles of 95°C for 15 seconds and combined annealing/extension at 68°C for 5 minutes, and final extension at 68°C for 10 minutes. Sanger sequence data was analyzed and assembled using Sequencher v 5.4.1 (Gene Codes Corp, Ann Arbor, MI). HBV surface antigen (HBsAg) subtyping was completed as previously described^26^ and the Geno2Pheno HBV online tool (Max Planck Institut Informatik, Germany; http://hbv.geno2pheno.org) was used to identify escape and resistance mutations.

### 2.3 Phylogenetic analysis

Sequences were merged with reference sequence alignments including 29 complete HBV genotype I genomes obtained from a comprehensive alignment generated from the Genbank database on 13 February 2018 (version 1.0 alignment from Bell et al^5^), plus eight additional published complete genome sequences^14^, ^15^, ^18^ in BioEdit v7.2.5.^27^ Neighbor‐joining HBV phylogenetic trees were generated as previously described,^26^ and trees were visualized using FigTree software (version 1.4.2; A. Rambaut, Institute of Evolutionary Biology, University of Edinburgh, Scotland). HBV‐I strains branching closely with other references were removed to generate a simpler tree for visualization in Figure 1B. Recombinant analysis was completed using Simplot (version 3.5.1) using a window of 400 basepairs (bp), step size of 20 bp, GapStrip on, 100 reps, Kimura two‐parameter, T/t 2.0, and neighbor‐joining settings. Genetic distances were calculated in BioEdit with complete genotype A to H reference strain sequence alignments generated from the Genbank database on 26 June 2017 (version 0.4 alignment from Bell et al^5^).

### 2.4 Serology testing

The HBV‐I specimen collected at the study entry day 0 was tested at a 1:500 dilution using ARCHITECT HBsAg Qualitative II, and the ARCHITECT CORE assays, and neat using the ARCHITECT HBeAg assay in combination with the ARCHITECT HBeAg Quantitative Calibrators (Abbott Diagnostics, Abbott Park, IL,)

### 2.5 Data availability

The HBV‐I genome has been deposited into GenBank under accession MH368022. Subgenomic HBV sequences for genotypes A, C, and F specimens have been deposited under accessions MH368023‐MH368033.

## 3 RESULTS

**Figure 2:**
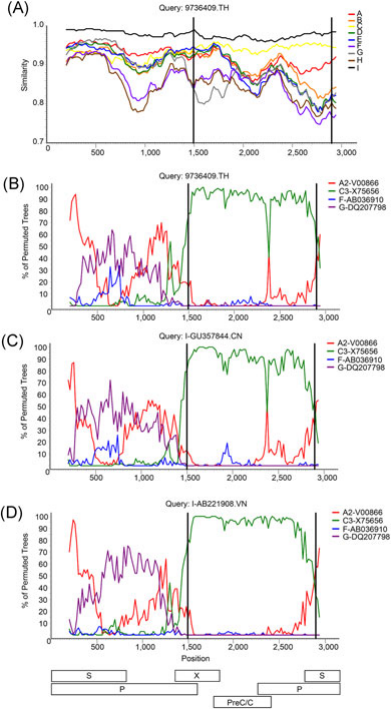
Recombinant analysis. A similarity plot generated in SimPlot with consensus sequences for each genotype is shown in panel (A). Bootscan plots comparing sample 9736409 (B), or HBV‐I strains from China (C), and Vietnam (D), to indicated reference strains are shown above a diagram of the HBV ORFs corresponding to the nucleotide positions indicated by the *x*‐axis. Breakpoints are indicated by a black line. HBV, hepatitis B virus; HBV‐I, hepatitis B virus genotype I; ORFs, open reading frames

Phylogenetic classification of SRT sequences obtained from plasma donations of 12 chronic HBV carriers enrolled in the iPrEx study identified genotype A1 (South Africa), A2 (United States), C1 (Thailand), F1 (Peru), F4 (Peru), and I (Thailand) infections (Figure 1A). Given the limited number of genome sequences available for HBV‐I infections, two additional overlapping amplicons were sequenced from the HBV‐I specimen to produce a complete genome of 3215 nucleotides. This specimen was collected in Chiang Mai, Thailand, 12 days before patient 9736409 began PrEP treatment, and subgenomic sequences generated from serial samples collected at later timepoints during PrEP treatment were all identical to the sequence obtained from the initial sample. Phylogenetic analysis of the complete genome indicated that the 9736409 sequence branched with other HBV‐I reference strains, but was basal to a set of reference strains from China (Figure 1B). SimPlot confirmed that the 9736409 sequence shared 95.1% to 99% similarity to a genotype I consensus sequence across the entire length of the genome (Figure 2A), and recombinant bootscan analysis indicated that it had a U/C recombinant pattern similar to other HBV‐I genomes from China and Vietnam (Figure 2B‐D),^8^, ^12^, ^14^, ^15^, ^18^, ^22^ with a genotype C region at nucleotide positions 1500 to 2900. Within the HBV surface antigen open reading frame (HBsAg ORF), the HBsAg subtype was determined to be *adw2* and an early termination mutation was identified at amino acid 216 (T801A). Likewise, an early stop codon was identified in the precore region of the 9736409 sequence at amino acid 28 (G1896A), which encodes the HBV e antigen (HBeAg). Importantly, no resistance mutations were found in the complete RT ORF as previously noted.^25^ The average nucleotide divergence of the 9736409 genome ranged from 7.66% (standard deviation [SD], 0.42%) for *n* = 1543 genotype C reference sequences to 14.27% (SD, 0.31%) for *n* = 17 genotype H reference sequences. In contrast, 9736409 was only 3.06% (SD, 0.52% ) divergent compared to *n* = 29 unique genotype I strains with complete genome sequences. The diluted HBV‐I specimen was HBsAg positive by ARCHITECT HBsAg Qualitative II (3269.79 S/CO), and anti‐HBc positive (8.25 S/CO). The same specimen was HBeAg negative when tested neat.

## 4 DISCUSSION

The unusual complex recombinants classified as HBV‐I include a growing number of sequences from individuals born in Asia, where HBV is highly endemic.^4^ The HBV‐I genotype has been defined by strains having 7% to 8% nucleotide divergence to the closest relative genotype C^8^, ^12^, ^14^, ^15^, ^18^, ^22^ and the new recombinant genome sequence presented here, 9736409, meets this criteria with a mean nucleotide divergence of 7.66% (SD, 0.42%) to genotype C reference sequences. Therefore, this sequence is classified as genotype I, making it the first HBV‐I strain identified from Thailand. With a HBsAg serotype of *adw2*, this specimen meets the criteria for the I1 subgenotype.^28^ Like other sequences classified as genotype I, 9736409 is a complex U/C recombinant with a similar recombination pattern and breakpoints (1500‐2900) as other strains from China and Vietnam (Figure 2).^8^, ^12^, ^14^, ^15^, ^18^, ^22^ Although other HBV‐I strains also have A‐ and G‐like regions^13^, ^14^, ^15^, ^16^, ^17^(Vietnam refs, Laos, India), these segments could not be classified in the 9736409 genome due to low bootstrap support (Figure 2). Notably, genotype G sequences are quite rare in Asia, with all reported strains coming primarily from the United States, France, and Germany.^4^ However, a genotype G/C recombinant (CU400) has been reported in Thailand,^29^ indicating that genotype G strains might be present in the region. Yet the 9736409 strain is unlikely to be closely related to the CU400 strain since the C‐regions of the two sequences have different breakpoints and the two strains only share 89% sequence identity.^29^ Consistent with previous HBV‐I genome analyses,^8^, ^13^, ^16^ the region of the 9736409 genome classified as genotype C branched basal to reference strains (Figure 1C). This suggests that the recombination events that resulted in HBV‐I did not occur recently, although in additional, analysis and sequences will be required to explore the origins of HBV‐I further. Likewise, the unclassifiable region from 1 to 1500 (Figure 2) is distinct from all other HBV genotypes (Figure 2),^8^ which further supports the identification of I as a separate genotype.

Examination of the amino acid sequences of the 9736409 open reading frames (ORFs) did not identify escape or resistance mutations in HBsAg or the polymerase. However, an early stop codon was identified in the HBsAg ORF at amino acid position 216 (T801A), which is a naturally occurring variant that results in a 10–amino‐acid truncation of the HBsAg ORF.^30^ While our direct amplicon sequencing method identified this variant as the major sequence present in 9736409, the possibility cannot be ruled out that a minor population of wildtype virus could produce full length HBsAg in this specimen. In a comparison of all 39 HBV‐I genomes with HBsAg and pre‐C coverage, only one other strain (LA.KF214679, G668A) carried a nonsense HBsAg mutation, although it is unknown whether HBsAg was detectable in this specimen.^13^ Similarly, a minority carried pre‐C nonsense mutations (*n* = 7; LA.KF214679, LA.KF214648, LA.KF214680, LA.FJ023665, LA.FJ023666, LA.FJ023672, and IN.EU835242), indicating that the nonsense mutations identified in the 9736409 genome are not representative of the entire genotype. Nonetheless, despite an early HBsAg stop codon and unclassifiable sequence in the HBsAg ORF, an HBsAg level of 34 456 IU/mL was detected by the ARCHITECT HBsAg assay (Abbott Diagnostics) and a viral load of 3.26 log IU/mL was detected by the m2000 RealTi*m*e HBV assay (Abbott Molecular Diagnostics, Des Plaines, IL) in the 9736409 specimen collected upon enrollment in the iPrEx study.^25^ Furthermore, the specimen was HBeAg negative as expected due to the precore amino acid 28 stop codon (G1896A). As HBV sequence diversity continues to expand, persistent global HBV surveillance remains essential to ensuring diagnostic tests can detect all strains regardless of their sequence or geographical location.
